# “Ick bin een Berlina”: dialect proficiency impacts a robot’s trustworthiness and competence evaluation

**DOI:** 10.3389/frobt.2023.1241519

**Published:** 2024-01-29

**Authors:** Katharina Kühne, Erika Herbold, Oliver Bendel, Yuefang Zhou, Martin H. Fischer

**Affiliations:** ^1^ Division of Cognitive Sciences, University of Potsdam, Potsdam, Germany; ^2^ School of Business FHNW, Brugg-Windisch, Brugg, Switzerland

**Keywords:** competence, dialect, human-robot interaction, robot voice, social robot, trust

## Abstract

**Background:** Robots are increasingly used as interaction partners with humans. Social robots are designed to follow expected behavioral norms when engaging with humans and are available with different voices and even accents. Some studies suggest that people prefer robots to speak in the user’s dialect, while others indicate a preference for different dialects.

**Methods:** Our study examined the impact of the Berlin dialect on perceived trustworthiness and competence of a robot. One hundred and twenty German native speakers (*M*_age_ = 32 years, *SD* = 12 years) watched an online video featuring a NAO robot speaking either in the Berlin dialect or standard German and assessed its trustworthiness and competence.

**Results:** We found a positive relationship between participants’ self-reported Berlin dialect proficiency and trustworthiness in the dialect-speaking robot. Only when controlled for demographic factors, there was a positive association between participants’ dialect proficiency, dialect performance and their assessment of robot’s competence for the standard German-speaking robot. Participants’ age, gender, length of residency in Berlin, and device used to respond also influenced assessments. Finally, the robot’s competence positively predicted its trustworthiness.

**Discussion:** Our results inform the design of social robots and emphasize the importance of device control in online experiments.

## 1 Introduction

### 1.1 Factors influencing robot’s acceptance

Social robots are becoming more common in various social aspects of human life, such as providing interpersonal care, tutoring, and companionship (Belpaeme et al., 2018; Bendel, 2021; Breazeal, 2017; Broadbent, 2017; Zhou and Fischer, 2019; for review, see e.g., Cifuentes et al., 2020; Woo et al., 2021; Henschel et al., 2021). Unlike most manufacturing or surgical robots, a social robot is designed to have a physical body and interact with humans in a way that aligns with human behavioral expectations (Bartneck and Forlizzi, 2004). Specifically, a humanoid robot is a type of a social robot with a body shape resembling a human, including a head, two arms, and two legs (Broadbent, 2017). According to Bendel (2021), social robots are sensorimotor machines created to interact with humans or animals. They can be identified through five key aspects. These are non-verbal interaction with living beings, verbal communication with living beings, representation of (aspects of or features of) living beings (e.g., they have an animaloid or a humanoid appearance or natural language abilities), proximity to living beings, and their utility or benefit for living beings. The assumption is that an entity is a social robot if four of these five dimensions are met. It can be hypothesized that the ability to speak and the voice used are likely to be among the central features of social robots. The present study focused on the role of speech to better understand social interactions with robots.

Which factors affect whether a person accepts a robot as a social interaction partner? Some of these factors include human-related aspects such as previous exposure to robots, the age and gender of the person interacting with robots (Broadbent et al., 2009; Kuo et al., 2009; Nomura, 2017; but also see Bishop et al., 2019; for a review, see Naneva et al., 2020). While it is generally observed that increased exposure to social robots corresponds to more favorable attitudes toward them, the evidence regarding age and gender as factors influencing acceptance is inconclusive. Previous studies suggested that older individuals and females tend to have less positive attitudes toward robots (Kuo et al., 2009; May et al., 2017). However, a systematic review (Naneva et al., 2020) contradicted this conclusion. According to this analysis, age and gender do not appear to have a significant impact on acceptance of social robots. Additionally, personality features might also play a role. According to Naneva et al. (2020), there is a positive correlation between acceptance of robots and the personality traits of agreeableness, extroversion, and openness, while conscientiousness and neuroticism do not appear to have any significant impact (Esterwood et al., 2021).

Apart from some human-related factors discussed above that could impact robot acceptance, many other factors that potentially influence human-robot interaction outcome concern the robot itself, including the purpose it is used for and its appearance. Whereas multiple studies demonstrated that users prefer human-like robots (Esposito et al., 2019; 2020), the systematic review by Naneva et al. (2020) could not find clear evidence for that. Here, we focus on some robot-related factors, in particular its voice, to motivate a novel research question, as will be reviewed in the next few paragraphs.

### 1.2 Anthropomorphism in robot design and its impact on interaction

People tend to ascribe human traits to non-human entities. There are two aspects to consider. Firstly, users attribute certain human behaviors to the robot by projecting their own expectations onto it. Secondly, individuals intentionally program the robot with human behaviors. Companies provide robots with a variety of physical appearances and voices that differ in gender, age, accent, and emotional expression, to cater to a wide range of needs and preferences of their users (Epley et al., 2007). An anthropomorphic robot design enables a more natural interaction with robots because people can rely on behaviors familiar from human-human interactions (Clodic et al., 2017). Moreover, a humanoid appearance results in more positive evaluation of the robot (Biermann et al., 2020).

### 1.3 Robot’s voice in trust and competence evaluation

To have a productive interaction, humans need to have confidence in and trust a social robot (Marble et al., 2004). Trust can influence the success of human-robot collaboration and determine future robot use (Freedy et al., 2007). In human-human interactions, trust has been the subject of extensive research (Dunning and Fetchenhauer, 2011). Crucially, multiple studies have indicated that trust does not necessarily result from a logical evaluation of the probabilities of different outcomes and benefits involved in a given situation. Rather, it seems to stem from non-rational factors, such as feelings and emotions. Factors that contribute to trust are linked to the attributes of both the person, the circumstances, and their interplay (Evans and Krueger, 2009; for review see Thielmann and Hilbig, 2015). In particular, being part of the same group can heighten trust levels (Evans and Krueger, 2009).

Trust in human-robot interaction is defined as “the attitude that an agent will help achieve an individual’s goals in a situation characterized by uncertainty and vulnerability” (Lee and See, 2004) or as “the reliance by an agent that actions prejudicial to their wellbeing will not be undertaken by influential others” (Hancock et al., 2011). These definitions imply that humans who trust a robot believe that it will not harm them or can be relied on in fulfilling tasks (Law and Scheutz, 2021).

Although numerous factors can impact trust in artificial agents (as demonstrated by Schaefer et al., 2016; Hancock et al., 2011 in their respective meta-analyses; for systematic review see Rheu et al., 2021; Law and Scheutz, 2021), the voice of a robot is considered one of the most critical factors in determining trust specifically related to robots.

In a questionnaire study conducted by Dautenhahn et al. (2005), most of the respondents expressed a desire for a robotic companion that can communicate in a way that is very similar to a human. Individuals also tended to get closer to a robot that had a human-like voice, in contrast to a robot with an artificially synthesized voice (Walters et al., 2008). Human-like voices were perceived as less uncanny and rated higher in terms of qualities such as sympathy, credibility, and trustworthiness (K. Kühne et al., 2020). Robots that had human-like voices were considered to be more efficient and were remembered more easily (Rodero, 2017). Finally, artificial agents with a human-like voice were perceived as more competent and credible (Sims et al., 2009; Fischer, 2021; Kim et al., 2022).

Competence is another attribute that is often intuitively assessed in everyday interactions (Kovarsky et al., 2013; Abele et al., 2021). The Behavioral Regulation Model defines confidence as the likelihood of task achievement (Ellemers et al., 2013). Alongside warmth, confidence underlies social evaluation and relies on such features as power, status, and resources (Rosenberg et al., 1968). In human-robot interaction, competence was one of the most important predictors of human preferences between different robot behaviors (Oliveira et al., 2019; Scheunemann et al., 2020). Also in evaluating competence, human-likeness in the robots’ appearance played a major role (Goetz et al., 2003; Kunold et al., 2023).

It is important to note that there is a significant association between competence and trust (Hancock et al., 2011; Kraus et al., 2018; Steain et al., 2019; Christoforakos et al., 2021). Individuals have greater trust in a robot when they perceive it to be more competent.

### 1.4 The uncanny valley phenomenon and its relation to a robot voice

One caveat in robot design is that incorporating too much human-likeness may result in the uncanny valley phenomenon. As shown by Mori (1970), the level of robot acceptance drops and a sense of eeriness or discomfort arises, once a certain level of human-like visual resemblance has been reached. Although there is currently no evidence of an uncanny valley for robotic voices (K. Kühne et al., 2020), it is premature to completely dismiss or exclude this possibility.

Assigning gender to a robot through appearance and voice can enhance its human-like qualities and influence its acceptance. For example, a female-sounding robot speaking in a higher tone received higher ratings for attractiveness and social competence (Niculescu et al., 2011; 2013). However, this effect can be influenced by the gender of the participants: Participants of the same gender as the robot’s given gender identify themselves more with the robot and feel closer to it (Eyssel et al., 2012). The process at work here is a tendency to favor those within one’s own group (in-group-bias; Tajfel and Forgas, 2000), which may extend to other facets of communication, such as a particular way of speaking or adopting regional language variations (Delia, 1975).

Another way to enhance the human-likeness of a robot’s voice is by incorporating an emotional tone or a particular dialect. Thus, robots with an emotional voice were found to be more likable (James et al., 2018). Researchers added a Scottish accent to Harmony, a customizable personal companion agent, in order to enhance her likability and charm (Coursey et al., 2019). Nevertheless, imparting a human dialect to a mechanically looking robot bears a risk of creating an uncanny valley effect (Mitchell et al., 2011). Therefore, we briefly review what is known about this mechanism of influence.

### 1.5 The impact of dialect-related social classifications and group identity

Interestingly, dialect-related social classifications and the sense of being part of a group based on accent or dialect are more robust than those resulting from gender or ethnicity (Kinzler et al., 2010). A dialect or accent refers to how individuals from diverse regions or social groups articulate words and phrases, leading to differences in their accent and speech patterns. While dialects and accents are interconnected, they are not identical. Dialects encompass a wider range of linguistic aspects, including vocabulary, grammar, and sentence structure, whereas accents primarily involve differences in pronunciation (Sikorski, 2005; for more detailed information on the topic of accent and dialect, see Planchenault and Poljak, 2021).

Evidence of the influence of dialect on the trust or competence of a robot is mixed. In general, according to the similarity-attraction theory, individuals tend to prefer artificial agents similar to themselves, for example, in terms of personality (Nass and Lee, 2000). However, similarity on a more superficial level, such as gender, was not found to predict trust (You and Robert, 2018).

In addition to identifying the speaker as a member of a particular geographical or national group, a dialect can also elicit favorable or unfavorable connotations and shape opinions about the speaker irrespective of the own group (H. Bishop et al., 2005). Listeners are sensitive to sociolinguistic information conveyed by a dialect or an accent. The standard language is typically viewed as prestigious and reliable, whereas regional accents tend to be regarded more unfavorably (H. Bishop et al., 2005; Tamagawa et al., 2011), so-called “accentism” (Foster and Stuart-Smith, 2023). However, certain languages may also have esteemed regional variations or dialects (H. Bishop et al., 2005).

Prejudices against dialects and their speakers cannot be ignored, as evaluations of dialects are often associated with evaluations of the corresponding population (Wiese, 2012). A meta-analysis by Fuertes et al. (2012) revealed that a spoken dialect is perceived as a sign of lower intelligence and social class. According to Wiese (2012), individuals who do not use the standard language are often viewed as linguistically incompetent. Furthermore, Fuertes et al. (2012) found that a spoken dialect can lower the perception of competence in general.

There are conflicting findings regarding the effects of different dialects on the perception of robots. On the one hand, imparting the standard language to a robot was shown to increase its trustworthiness and competence (Torre and Maguer, 2020). As an example, only around 4% of Torre and Maguer’s (2020) participants wanted the robot to have the same accent as they had, whereas 37% preferred a robot speaking the Standard Southern British English. Similar findings were obtained by Andrist et al. (2015): More native Arabic speakers complied with the robots who were speaking standard Arabic. For the dialect-speaking robot, the compliance depended on other factors. Namely, robots speaking with both high knowledge and high rhetorical ability were complied with more. Another study found that a synthetic agent with Austrian standard accent was perceived as possessing higher levels of education, trustworthiness, competence, politeness, and seriousness (Krenn et al., 2017).

On the other hand, robots speaking a dialect, in this case, Franconian, were rated as more competent (Lugrin et al., 2020). Unlike in Torre and Maguer (2020), the evaluation of competence depended on the participants’ own performance in the dialect. Those who spoke in dialect more frequently rated the dialect-speaking robot as more competent. In contrast to that, V. Kühne et al. (2013) found that participants liked a dialect-speaking robot more, irrespective of their own dialect performance. In the same vein, a robot was accepted in Norwegian hospitals more when it spoke the Trøndersk dialect (Søraa and Fostervold, 2021). This preference could have been impacted by the comfortable and pleasant connotation conveyed by the Trøndersk dialect. To embrace these discrepancies, there is currently an ongoing project to develop an optimal language or accent for an artificial agent to speak (Foster and Stuart-Smith, 2023).

In summary, standard language-speaking robots were perceived as more trustworthy or likable presumably due to the in-group bias and accentism, while according to other studies, participants preferred robots that spoke with a dialect. However, the preference for dialect-speaking robots was often influenced by human-related factors, namely, the participants’ proficiency or performance in that dialect (Lugrin et al., 2020).

Most of the research on the utilization of dialect in robots has been conducted in Anglo-Saxon countries (Früh and Gasser, 2018). As for German-speaking countries, V. Kühne et al. (2013) found that a Rhine-Ruhr dialect-speaking virtual robot was perceived as more likable. Another study by Früh and Gasser (2018) also reports more positive attitudes toward a dialect-speaking care robot Lio in Switzerland. Importantly, in Switzerland, a dialect serves strongly as a means of social demarcation. However, a most recent study with a service robot Pepper in a hotel context showed that using the local dialect did not affect robot acceptance and attitudes (Steinhaeusser et al., 2022). The study was conducted online and participants speaking the Flanconian dialect vs. standard German were randomly assigned to the dialect or standard language conditions. While there was a non-significant tendency for individuals who spoke a dialect to have a more negative attitude toward a robot that used that same dialect, this could potentially be attributed to the use of Pepper’s text-to-speech plugin to synthesize the dialect and accent. People with a local accent may have been more likely to notice any mistakes or errors in the robot’s synthesized speech, which could in turn have influenced their attitudes towards it.

To address the inconsistencies reviewed above, we conducted an online study among Berlin and Brandenburg residents in order to investigate the relationship between the participants’ proficiency and performance in the Berlin dialect and their trust in a robot, and the robot’s competence evaluation.

### 1.6 The present study

From 1500 onwards, the Berlin dialect emerged as a unique local language variety, replacing Low German in the region. The Berlin dialect is associated with the working class and often portrayed as a proletarian language by media figures who depict it as a dialect spoken by simple, but likable people. Additionally, the Berlin dialect is intentionally employed as a stylistic choice to establish a sense of closeness with a specific audience, as observed in its written representation in daily newspapers (Wiese, 2012). Specific features of the Berlin dialect can be found in Stickel (1997).

Dialect *proficiency* means the self-evaluated ability to speak the dialect, whereas dialect *performance* denotes the frequency with which the participants speak the dialect. We formulated six hypotheses for our study. The first hypothesis was that the standard German-speaking robot would be trusted more and evaluated as more competent than the dialect-speaking robot (H1). The next two hypotheses posited that participants with (H2) higher dialect proficiency and (H3) higher dialect performance would trust the robot more than those with lower dialect proficiency and performance. The fourth and fifth hypotheses were that participants with (H4) higher dialect proficiency and (H5) higher dialect performance would evaluate the robot’s competence higher than those with lower dialect proficiency and performance. Finally, we expected that the robot’s competence would predict the trust ratings (H6). Hypotheses H2—H6 were tested independently for the dialect-speaking robot (H2a, H3a, H4a, H5a, H6a) and the standard German-speaking robot (H2b, H3b, H4b, H5b, H6b) as alternatives. We tested these formulated hypotheses in an online experiment with German-speaking participants using the NAO robot.

## 2 Materials and methods

### 2.1 Participants and procedure

The experiment was programmed and run using the online Gorilla Experiment Builder research platform (Anwyl-Irvine et al., 2020) and lasted approximately 30 min. The participants were recruited via the subject pool system SONA at the University of Potsdam. All the participants submitted their informed consent at the beginning of the experiment by clicking the corresponding checkbox and were reimbursed with course credits for their participation. They were instructed to first watch a video and then answer the survey questions honestly and spontaneously. The type of the video (Berlin dialect or Standard German) was counterbalanced between participants. After the survey, the participants were asked to fill in a demographic questionnaire, including questions about their age, gender, native language, dialect proficiency, dialect performance, and duration of residence in Berlin. Finally, participants were debriefed and given a link to enter their internal subject pool ID for receiving a credit.

The study was conducted in accordance with the guidelines laid down in the Declaration of Helsinki and in compliance with the ethics policy of the University of Potsdam. No explicit approval was needed because the methods were standard. There were no known risks and participants gave their informed consent. The study and the procedure were already evaluated by professional psychologists to be consistent with the ethical standards of the German Research Foundation, including written informed consent and confidentiality of data as well as personal conduct.

An *a priori* power analysis was conducted using G*Power (Faul et al., 2007) to determine the minimum sample size required to test the study hypothesis. Results indicated the required sample size to achieve 80% power for detecting a medium effect, at a significance criterion of α = .05, was *N* = 68 per robot group for linear regression with two predictors (*N* = 136 in total).

### 2.2 Stimuli materials

**FIGURE 1:**
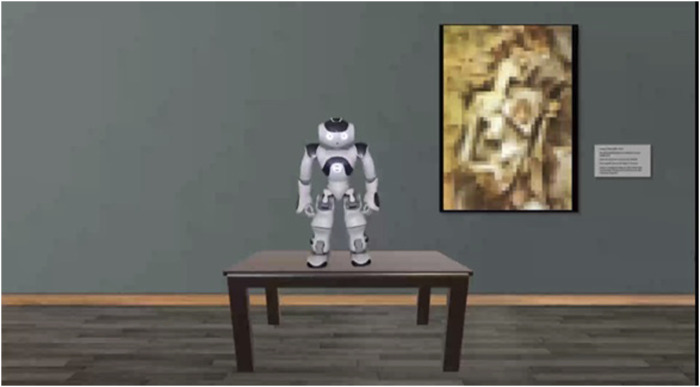
Screenshot of the Video Footage used *Note:* The artwork was pixelated in the videos to protect copyright. It is the painting *Girl with a Mandolin* by Pablo Picasso (1910).

We used a video lasting 31 s, showcasing the humanoid robot NAO (Aldebaran—SAS)^1^. In the video, the robot was positioned on a table and was in motion while providing details about a painting situated in the top right portion of the wall. The painting was pixelated to avoid copyright infringement. A snapshot from the video is depicted in Figure 1.

The robot in the video used a male human voice to speak. The speech was recorded twice by the same speaker—once in standard German and once in the Berlin dialect. The transcription can be found in Supplementary Materials.

We opted to use a human voice based on earlier studies, which indicated that people prefer less robotic-sounding voices as they feel more at ease while listening to them (Dong et al., 2020; K; Kühne et al., 2020). Natural human voices are generally perceived as more trustworthy and competent compared to synthetic voices (Craig and Schroeder, 2017; Kühne et al., 2020; Sims et al., 2009). Moreover, listening to a synthetic voice can increase one’s cognitive load (Francis and Nusbaum, 2009; Simantiraki et al., 2018) which, in its turn, can lead to trust misplacement (Duffy and Smith, 2014).

We selected a male voice because research suggests that NAO is more commonly associated with a male voice (Behrens et al., 2018). The stimuli can be found at: https://osf.io/pfqg6/.

### 2.3 Measures

#### 2.3.1 Independent variables

##### 2.3.1.1 Demographic factors

The following demographic factors were measured: age, gender, native language, and duration of residence in Berlin (in years).

##### 2.3.1.2 Dialect proficiency

The dialect proficiency was measured using a single item: “How well can you speak the Berlin dialect?”. The answers were given on a seven-point Likert scale from 1 (Not at all) to 7 (Very well).

##### 2.3.1.3 Dialect performance

The dialect performance was measured using a single item: “In everyday life, I usually speak the Berlin dialect”. The answers were given on a seven-point Likert scale from 1 (Does not apply at all) to 7 (Applies totally).

##### 2.3.1.4 Device type

Device type was automatically measured by the experiment system as “mobile”, “tablet”, or “computer”.

#### 2.3.2 Dependent variables

##### 2.3.2.1 Trust

We used the *Scale of Trust in Automated Systems* (Jian et al., 2000) to access the level of trust participants had toward the robot featured in the video. The scale consists of 12 items, measured on a seven-point Likert scale from 1 (Do not agree at all) to 7 (Fully agree), and was specifically designed to measure trust towards automated systems, such as robots. To suit the study’s German setting, the items were translated into German, and the word “system” in each item was replaced with “robot” to better relate to the robot shown in the video. Sample items were: “I can trust the robot” („*Ich kann dem Roboter vertrauen*”); “The robot is dependable” (“*Der Roboter ist verlässlich*”). Supplementary Table S1 displays the original items and their corresponding German translations. Additionally, an extra attention-testing item was added to the scale, which instructed participants to choose response option 7 (Fully agree) as their response.

##### 2.3.2.2 Competence

We used the *Robotic Social Attribute Scale* (RoSAS) (Carpinella et al., 2017) to measure the competence evaluation of the featured robot. The scale consists of 6 items, measured on a seven-point Likert scale from 1 (Do not agree at all) to 7 (Fully agree). Sample items were: “The robot is interactive” (“*Der Roboter ist interaktiv*”); “The robot is knowledgeable” (“*Der Roboter ist sachkundig*”). Supplementary Table S2 displays the original items and their corresponding German translations. Additionally, an extra attention-testing item was added to the scale, which instructed participants to choose the response option 1 (“Do not agree at all”) as their response.

### 2.4 Sample and data pre-processing

One hundred and thirty-seven participants (94 females, 41 males, 2 non-binary), *Mean* age = 33 years, *SD* = 14 years) took part in the experiment. Eight participants were excluded from the analysis because their native language was not German. Nine participants were further excluded from the analysis because they failed the attention test items in both scales. This yielded the ultimate sample size *N* = 120 (*Mean* age = 32 years, *SD* = 12 years; 81 female, 38 male, 1 non-binary). Additionally, data from the trust items of two participants and data from the competence items of three participants were excluded because they failed the attention test items in the respective scale. The remaining data of these five participants was still used.

Data preparation and analyses were done using Microsoft^®^ Excel^®^ for Microsoft 365 and SPSS Version v.29 software package. Figures were built in R (R Core Team, 2020). The normality of the data distribution was confirmed using a *Kolomogorov-Smirnov* test. Before conducting the multiple regression analysis, the distributional assumptions for the multiple regression were assessed^2^. The regression analysis treated the gender category of “non-binary” as missing data.

## 3 Analysis and results

### 3.1 Trust

First, we employed a two-tailed independent samples *t*-test to examine the level of trust between the dialect-speaking robot and the standard German-speaking robot in all participants. Even though there was a minor trend in favor of trusting the standard German-speaking robot more (*M* = 4.716, *SD* = 1.259) than the dialect-speaking one (*M* = 4.591, *SD* = 1.056), this difference was not statistically significant (*t* (116) = −0.583, *p* = .561). Thus, we failed to confirm H1a. Participants did not trust the standard German-speaking robot significantly more than the dialect-speaking robot.

To examine if participants with higher dialect proficiency would trust the dialect-speaking robot more than those with lower dialect proficiency, we conducted a multiple regression analysis, using the enter method. In the first step, we added only dialect proficiency as predictor. In the second step, we added control variables: age, gender, duration of residence in Berlin, and device type. In line with the H2a hypothesis, only dialect proficiency explained a significant amount of the variance in the value of trust in the dialect-speaking robot (*β* = .272, *t* (60) = 2.189, *p* < .05, *F* (1, 60) = 4.792, *R* ^2^ = .074, *R* ^2^_Adjusted_ = .059). The dialect-speaking robot was more trusted by participants who were more proficient in the Berlin dialect.

We conducted another multiple regression analysis to see if participants with higher dialect performance would trust the dialect-speaking robot more than those with lower dialect performance. Again, in the first step, we added only dialect performance as predictor. In the second step, we added control variables: age, gender, duration of residence in Berlin, and device type. Contrary to the H3a hypothesis, dialect performance was not a significant predictor of trust in the dialect-speaking robot (*β* = .208, *t* (60) = 1.646, *p* = .105, *F* (1, 60) = 2.711, *R* ^2^ = .043, *R* ^2^_Adjusted_ = .027). Neither of the control variables contributed to the variance of trust neither.

In summary, for the dialect-speaking robot, only dialect proficiency was a significant predictor of trust. We confirmed H2a and failed to confirm H3a.

Further, we conducted a multiple regression analysis to test if participants with higher dialect proficiency would trust the standard German-speaking robot more than those with lower dialect proficiency. Again, using the enter method, in the first step, we added only dialect proficiency as predictor. In the second step, we added control variables: age, gender, duration of residence in Berlin, and device type.

Contrary to the H2b hypothesis, dialect proficiency did not explain the value of trust in the standard-speaking robot (*β* = .086, *t* (53) = 0.628, *p* = .533, *F* (1, 53) = 0.394, *R* ^2^ = .007, *R* ^2^_Adjusted_ = −.011). However, age, gender, duration of residence in Berlin, and device type were significant predictors of trust. The standard German-speaking robot was more trusted by individuals who were older, female, had a shorter duration of residence in Berlin, and used a computer device for watching the experimental videos.

Finally, we conducted another multiple regression to examine if participants with higher dialect performance would trust the standard German-speaking robot more than those with lower dialect performance. In the first step, we added only dialect performance as predictor. In the second step, we added control variables: age, gender, duration of residence in Berlin, and device type. Contrary to the H3b hypothesis, dialect performance was not a significant predictor of trust in the standard-speaking robot (*β* = .043, *t* (53) = 0.312, *p* = .757, *F* (1, 53) = 0.097, *R* ^2^ = .002, *R* ^2^_Adjusted_ = −.017).

In summary, for the standard German-speaking robot, age, gender, duration of residence in Berlin, and device type were significant predictors of trust, when together in model with dialect proficiency. We found no evidence for H2b and H3b.

The results are summarized in Table 1 and Table 2.

**TABLE 1:** Results of the Regression Analysis on the Outcome Variable Trust with Dialect Proficiency as Predictor.

Dialect-speaking robot						Standard German-speaking robot			
Model		*β*	*SE*	*t*	*p*	*β*	*SE*	*t*	*p*
1	Constant		0.227	18.401	<.001		0.315	14.457	<.001
	Proficiency	**.272**	**0.061**	**2.189**	**< .05**	.086	0.079	0.628	.533
*R* ^*2*^		.074				.007			
*R* ^*2*^_*Adjusted*_		.059				−.011			
*p*		<.05				.533			
2	Constant		0.532	7.913	<.001		0.561	7.626	<.001
	Proficiency	.345	0.094	1.824	.074	.308	0.101	1.768	.083
	Age	.174	0.013	1.210	.231	**.426**	**0.015**	**2.916**	**<.05**
	Gender	−.144	0.306	−1.125	.265	**−.319**	**0.325**	**−2.528**	**<.05**
	Duration	−.179	0.078	−0.917	.363	**−.471**	**0.091**	**−2.537**	**<.05**
	Device	.082	0.266	0.645	.522	**.428**	**0.316**	**3.412**	**<.001**
*R* ^*2*^		.119				.325			
*R* ^*2*^_*Adjusted*_		.040				.356			
*p*		.200				<.001			

Note: Dialect-speaking robot *N* = 63. Standard-speaking robot *N* = 57.

Method: enter. Significant results are marked in bold.

**TABLE 2:** Results of the Regression Analysis on the Outcome Variable Trust with Dialect Performance as Predictor.

Dialect-speaking robot						Standard German-speaking robot			
Model		*β*	*SE*	*t*	*p*	*β*	*SE*	*t*	*p*
1	Constant		0.206	21.069	<.001		0.272	17.127	<.001
	Performance	.208	0.090	1.646	.105	.043	0.095	0.312	.757
*R* ^*2*^		.043						.002	
*R* ^*2*^_*Adjusted*_		.027						−.017	
*p*		.105						.757	
2	Constant		0.544	7.742	<,001		0.563	7.613	<.001
	Performance	.142	0.108	0.933	.355	.259	0.107	1.683	.099
	Age	.174	0.014	1.170	.247	**.430**	**0.016**	**2.924**	**<.05**
	Gender	−.129	0.313	−0.984	.329	**−.313**	**0.324**	**−2.480**	**<.05**
	Duration	.004	0.064	0.025	.980	**−.420**	**0.084**	**−2.462**	**<.05**
	Device	.074	0.271	0.568	.572	**.475**	**0.311**	**3.844**	**<.001**
*R* ^*2*^		.081						.321	
*R* ^*2*^_*Adjusted*_		−.001						.252	
*p*		.434						<.05	

Note: Dialect-speaking robot *N* = 63. Standard-speaking robot *N* = 57.

Significant results are marked in bold.

**FIGURE 2:**
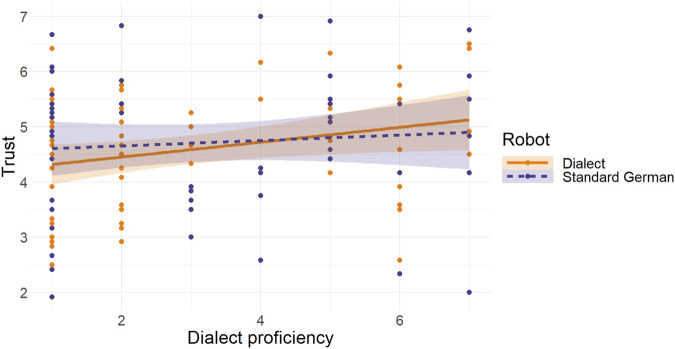
Regression Analysis for Dialect Proficiency as a Predictor of Trust in the Standard German-speaking and the Dialect-speaking Robot *Note:* The orange solid line represents the regression slope for the dialect-speaking robot. The dark blue long dashed line represents the regression slope for the standard German-speaking robot.

Figure 2 presents a visual summary of the outcomes obtained from regression analyses that assessed how dialect proficiency predicted trust in both the standard German-speaking and dialect-speaking robot.

### 3.2 Competence

Again, we used a two-tailed independent samples *t*-test to examine the level of competence between the dialect-speaking robot and the standard German-speaking robot in all participants. The findings were similar for the evaluation of trust. While there was a descriptive tendency to rate the standard German-speaking robot as more competent (*M* = 3.831, *SD* = 0.947) than the dialect-speaking robot (*M* = 3.777, *SD* = 0.999), the difference was not statistically significant (*t* (115) = −0.303, *p* = .763). Thus, we failed to confirm H1b. Participants did not evaluate the standard German-speaking robot as significantly more competent than the dialect-speaking robot.

To examine if participants with higher dialect proficiency would evaluate the dialect-speaking robot as more competent than those with lower dialect proficiency, we again conducted a multiple regression using the enter method. In the first step, we added only dialect proficiency as predictor. In the second step, we added control variables: age, gender, duration of residence in Berlin, and device type. Contrary to the H4a hypothesis, dialect proficiency was not a significant predictor of competence in the dialect-speaking robot (*β* = .047, *t* (60) = 0.363, *p* = .718, *F* (1, 60) = 0.131, *R* ^2^ = .002, *R* ^2^_Adjusted_ = −.014).

To examine if participants with higher dialect performance would evaluate the dialect-speaking robot as more competent than those with lower dialect performance, we again conducted a multiple regression using the enter method. In the first step, we added only dialect performance as predictor. In the second step, we added control variables: age, gender, duration of residence in Berlin, and device type. Again, counter to the H5a hypothesis, dialect performance was not a significant predictor of competence in the dialect-speaking robot (*β* = −.002, *t* (60) = −0.019, *p* = .985, *F* (1, 60) = 0.000, *R* ^2^ = .000, *R* ^2^_Adjusted_ = −.017).

Neither of the control variables contributed to the variance of competence.

In summary, for the dialect-speaking robot, neither dialect proficiency nor dialect performance, or any control variable was significant predictor of competence. We found no evidence for H4a and H5a.

Further, to examine if participants with higher dialect proficiency would evaluate the standard German-speaking robot as more competent than those with lower dialect proficiency, we conducted a multiple regression using the enter method. In the first step, we added only dialect proficiency as predictor. In the second step, we added control variables: age, gender, duration of residence in Berlin, and device type. Contrary to the H4b hypothesis, dialect proficiency alone was not a significant predictor of competence in the standard-speaking robot (*β* = .086, *t* (52) = 0.623, *p* = .536, *F* (1, 52) = 0.389, *R* ^2^ = .007, *R* ^2^_Adjuste_d = −.012). However, when controlled for age, gender, duration of residence in Berlin, and device type, it did explain a reliable amount of variance in the value of competence, together with duration of residence in Berlin ( *β* = .695, *t* (48) = 3.463, and *β* = −.824, *t* (48) = −3.735, respectively, *p* < .001, *F* (5, 48) = 4.634, *R* ^2^ = .326, *R* ^2^_Adjusted_ = .255). age, gender, and device type did not contribut to the final model.

To see if participants with higher dialect performance would evaluate the standard German-speaking robot as more competent than those with lower dialect performance, we conducted a multiple regression using the enter method. In the first step, we added only dialect performance as predictor. In the second step, we added control variables: age, gender, duration of residence in Berlin, and device type.

Contrary to the hypothesis H5b, dialect performance alone was not a significant predictor of competence in the standard German-speaking robot (*β* = .051, *t* (52) = 0.365, *p* = .717, *F* (1, 52) = 0.133, *R* ^2^ = .003, *R* ^2^_Adjusted_ = −.017). However, when controlled for age, gender, duration of residence in Berlin, and device type, it did explain a reliable amount of variance in the value of competence, together with duration of residence in Berlin and device type ( *β* = .410, *t* (48) = 2.433; *β* = −.529, *t* (48) = −2.768; and *β* = .281, *t* (48) = 2.188 respectively, *p* < .05, *F* (5, 48) = 3.193, *R* ^2^ = .250, *R* ^2^_Adjusted_ = .171). age and gender did not contribute to the final model.

In summary, for the standard German-speaking robot both dialect proficiency and dialect performance were significant predictors of competence, but only when controlled for age, gender, duration of residence in Berlin, and device type. Hypotheses H4b and H5b could be partially confirmed. Duration of residence in Berlin and device type were also reliable predictors of competence for the standard German-speaking robot.

The results are summarized in Table 3 and Table 4.

**TABLE 3:** Results of the Regression Analysis on the Outcome Variable Competence with Dialect Proficiency as Predictor.

Dialect-speaking robot						Standard German-speaking robot			
Model		*β*	*SE*	*t*	*p*	*β*	*SE*	*t*	*p*
1	Constant		0.226	16.413	<.001		0.234	15.792	<.001
	Proficiency	.047	0.061	0.363	.718	.086	0.059	0.623	.536
*R* ^*2*^		.002				.007			
*R* ^*2*^_*Adjusted*_		−.014				−.012			
*p*		.718				.536			
2	Constant		0.502	9.231	<.001		0.421	9.798	<.001
	Proficiency	.293	0.087	1.592	.117	**.695**	**0.086**	**3.463**	**<.001**
	Age	−.216	0.012	−1.489	.142	.125	0.012	0.807	.423
	Gender	−.152	0.289	−1.191	.239	−.199	0.250	−1.529	.133
	Duration	−.222	0.073	−1.131	.263	**−.824**	**0.083**	**−3.735**	**<.001**
	Device	.079	0.253	0.623	.536	.192	0.233	1.562	.125
*R* ^*2*^		.121				.326			
*R* ^*2*^_*Adjusted*_		.042				.255			
*p*		.193				<.05			

Note: Dialect-speaking robot *N* = 63. Standard-speaking robot *N* = 57.

Significant results are marked in bold.

**TABLE 4:** Results of the Regression Analysis on the Outcome Variable Competence with Dialect Performance as Predictor.

Dialect-speaking robot						Standard German-speaking robot			
Model		β	SE	t	p	β	SE	t	p
1	Constant		0.197	19.167	<.001		0.206	18.243	<.001
	Performance	−.002	0.082	−0.019	.985	.051	0.072	0.365	.717
*R* ^*2*^	.000						.003		
*R* ^*2*^_*Adjusted*_		−.017					−.017		
*p*		.985					.717		
2	Constant		0.509	9.190	<.001		0.444	9.389	<.001
	Performance	.139	0.099	0.890	.377	**.410**	**0.087**	**2.433**	**<.05**
	Age	−.246	0.012	−1.619	.111	.005	0.012	0.029	.977
	Gender	−.132	0.294	−1.017	.314	−.122	0.256	−0.915	.365
	Duration	−.068	0.060	−0.423	.674	**−.529**	**0.072**	**−2.768**	**<.05**
	Device	.079	0.257	0.614	.542	**.281**	**0.244**	**2.188**	**<.05**
*R* ^*2*^		.094				.250			
*R* ^*2*^_*Adjusted*_		.013				.171			
*p*		.341				<.05			

Note: Dialect-speaking robot *N* = 63. Standard-speaking robot *N* = 57.

Method: enter. Significant results are marked in bold.

**FIGURE 3:**
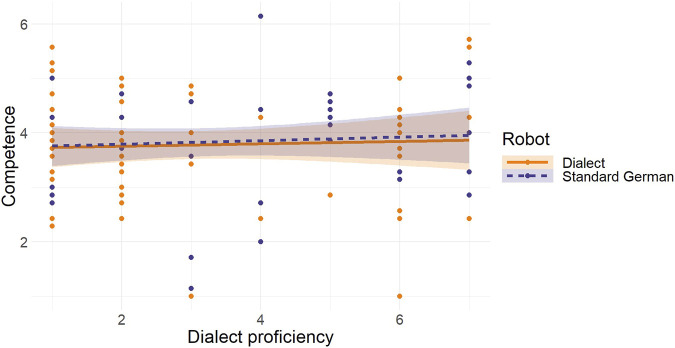
Regression Analysis for Dialect Proficiency as a Predictor of Competence in the Standard German-speaking and the Dialect-speaking Robot Note: The orange solid line represents the regression slope for the dialect-speaking robot. The dark blue long dashed line represents the regression slope for the standard German-speaking robot.

Figure 3 presents a visual summary of the outcomes obtained from regression analyses that assessed how dialect proficiency predicted competence in both the standard German-speaking and dialect-speaking robot.

### 3.3 Association between robot’s competence and trust

**FIGURE 4:**
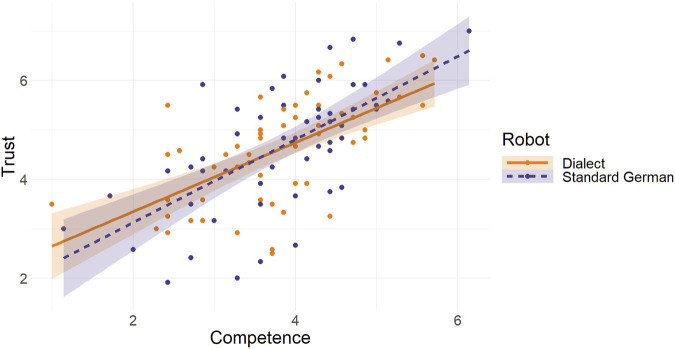
Regression Analysis of Competence as a Predictor of Trust Note: The orange solid line represents the regression slope for the dialect-speaking robot. The dark blue long dashed line represents the regression slope for the standard German-speaking robot.

Lastly, we sought to determine if the evaluation of a robot’s competence could predict the degree of trust that was placed in the robot. Indeed, for both the dialect-speaking robot (*β* = .631, *t* (59) = 6.249, *F* (1, 59) = 39.049, *p* < .001, *R* ^2^ = .398, *R* ^2^_Adjusted_ = .388) and the standard German-speaking robot (*β* = .646, *t* (52) = 6.096, *F* (1, 52) = 37.164, *p* < .001, *R* ^2^ = .417, *R* ^2^_Adjusted_ = .406), competence was a significant predictor of trust. Both H6a and H6b could be confirmed. Figure 4 presents a visual representation of the outcomes of the regression analyses.

The data set and the analysis script can be found at: https://osf.io/pfqg6/.

## 4 Discussion

### 4.1 Proficiency and performance in the Berlin dialect and evaluation of competence and trust

Our study investigated verbal aspects of human robot interaction quality. Specifically, we examined the association between participants’ proficiency and performance in the Berlin dialect and their evaluation of competence and trust in a NAO robot that spoke either with or without this dialect. The study was conducted online, and dialect proficiency was defined as the self-evaluated ability to speak the Berlin dialect, while dialect performance referred to the frequency of dialect used by the participants.

In general, although the difference in trust and competence ratings were not significant, our findings tend to be consistent with previous studies conducted by Torre and Maguer (2020) and Andrist et al. (2015) which also found that people preferred a robot that speaks in standard language. This is in line with the overall research suggesting that individuals who speak the standard language are perceived as more competent (Fuertes et al., 2012). However, our findings are contradictory to the results of V. Kühne et al. (2013) and Früh and Gasser (2018) where a robot speaking in dialect was viewed more positively. It is essential that their experiments were conducted in Switzerland, as the local dialect plays a crucial role, in distinguishing insiders from outsiders. Further, similar to Lugrin et al. (2020) we demonstrated that participants’ ratings of the robot’s trust and competence were influenced by their own proficiency in the dialect, but our study provided more nuanced results.

Importantly, as expected, the competence of the robot significantly predicted trust. Namely, the more competent the robot was rated by the participants, the more they trusted it. This is in line with previous research (Hancock et al., 2011; Kraus et al., 2018; Steain et al., 2019; Christoforakos et al., 2021). Competence is perceived as an ability to carry out behavioral intentions (Kulms and Kopp, 2018). Being a positive quality, it creates a more favorable impression of the trustee. As a major dimension of social cognition postulated by the Stereotype Content Model, competence has been observed to foster the establishment of trust in interactions between humans (Fiske et al., 2007). Also according to another model, competence and benevolence of the trustee are positively related to trust (Mayer et al., 1995). Thus, we report evidence indicating that social mechanisms observed in human-human interactions can be transferred to human-robot interactions.

In the following paragraphs we will discuss the findings in detail. In the first place, although there was a slight trend of higher trust and competence evaluation for the standard German-speaking compared to the dialect-speaking robot for all participants, the difference was not statistically significant. The standard German-speaking robot and the dialect-speaking robot received largely comparable ratings in terms of both competence and trustworthiness.

Nevertheless, there were systematic differences in ratings between the two robots. Consider first the ratings obtained for the dialect-speaking robot. For the dialect-speaking robot, only dialect proficiency was a significant predictor of trust, with individuals who considered themselves more proficient in speaking the Berlin dialect having higher levels of trust. The other predictors (dialect performance, age, gender, duration of residence, and device type) did not have a significant contribution to the final statistical model of the ratings on trust. Our analysis for the outcome variable competence showed no significant predictors. Dialect proficiency, dialect performance, age, gender, duration of residence, and device type did not significantly contribute to the final model of participants’ rating. Thus, for the dialect-speaking robot, only one reliable association was found, namely, that between dialect proficiency and the trust in robots. The more proficient the participants were in the Berlin dialect, the more they trusted the dialect-speaking NAO, exactly in the sense of the similarity-attraction theory (Nass and Lee, 2000). None of the factors were found to be predictive of the level of robot’s competence.

For the standard German-speaking robot, the findings were more complex. We found that the final model included age, gender, duration of residence, and device type as significant predictors of trust, but only when included into the model together with dialect proficiency. Individuals who were older, female, had a shorter duration of residence in Berlin, and used a computer device for watching the experimental videos were found to trust the standard German-speaking robot more. Dialect performance did not make a significant contribution to the model.

Finally, dialect proficiency, dialect performance, duration of residence, and device type were significant predictors of competence, indicating that those who were more proficient in speaking the Berlin dialect, spoke it more often, had a shorter duration of residence in Berlin, and used a computer device for watching the experimental videos found the standard German-speaking robot more competent.

For the standard German-speaking robot, general factors such as age and gender appeared to be predictive of the trust level, while the participants’ dialect proficiency and performance only played a role in the evaluation of competence. This finding collaborates with earlier research reporting the importance of demographic factors on robot’s perception (Naneva et al., 2020). Similar to results obtained by K. Kühne et al. (2020), female participants evaluated the robot as more trustworthy. In comparison to that research, however, we found that, as participants’ age increased, their trust in the standard German-speaking robot also increased. In conclusion, again following the principles of the similarity-attraction theory (Nass and Lee, 2000), participants who had been living in Berlin for a shorter period, presumably were less likely to be influenced by the Berlin dialect, were more likely to trust the robot that spoke in standard German and found it more competent.

It is noteworthy that not dialect performance as a relatively objective and quantitative measure of a dialect usage but dialect proficiency, a subjective and qualitative evaluation of one’s dialect mastery, predicted the robot’s perceived trustworthiness. The ability to speak a dialect can be integral to one’s self-image and contribute to the identification of oneself with a particular group or set of qualities. According to recent research, it is so-called self-essentialist reasoning, that is beliefs about the essence of one’ self, that underly the similarity-attraction effect (Chu et al., 2019). This reasoning focuses more on what one is and not on what one does; it is a personal characteristic that tends to be stable rather than situational or temporary in nature.

On a side note, participants who watched the video on a PC rated the standard German-speaking robot as more trustworthy and more competent, compared to participants working on a tablet or a mobile phone. This result indicates that, when examining human-robot interaction through video or audio stimuli, it is important to consider and control for the experimental device used. Possible reasons for the observed difference include different testing situations, such as doing the experiment at home on a PC or “on the go” on a mobile phone, which could have resulted in different distractions and response criteria, or differences in information processing on different screens (cf. Sweeney and Crestani, 2006; Wickens and Carswell, 2021). These factors could have potentially led to increased cognitive load on smaller screens and, consequently, to trust misplacement (Duffy and Smith, 2014).

### 4.2 Limitations of the study

It is worth noting that various intervening factors could have influenced our study. First, choosing a male voice might have affected the overall outcomes. Unlike in human-human interactions (Bonein and Serra, 2009; Slonim and Guillen, 2010), prior studies have shown that virtual assistants or robots with a male voice are generally viewed as more competent (Powers and Kiesler, 2006; Ernst and Herm-Stapelberg, 2020) and trustworthy (Behrens et al., 2018), although these ratings can be context-dependent (Andrist et al., 2015; Kraus et al., 2018). On the contrary, other recent research indicates that a female voice agent may be viewed as more likable, competent, or intelligent (Vega et al., 2019; Dong et al., 2020). ‬‬‬‬‬‬‬‬‬‬‬‬‬‬‬‬‬‬‬‬‬‬‬‬‬‬‬‬‬‬‬‬‬‬‬‬‬‬‬‬‬‬‬‬‬‬‬‬‬‬‬‬‬‬‬‬‬‬‬‬‬‬

Second, due to social identification, people tend to rate voices of the same gender as more trustworthy (Crowelly et al., 2009) and perceive more psychological closeness to them (Eyssel et al., 2012). However, our research did not find evidence for this when using male voice stimuli exclusively. To resolve these contradictory results, more studies utilizing both male and female voices are necessary.‬‬‬‬‬‬‬

Third, dialects carry distinct connotations within German-speaking countries (cf. H. Bishop et al., 2005). For instance, the Berlin dialect is often associated with a lower socioeconomic class or working class (Stickel, 1997), whereas the Bavarian dialect is often viewed as more prestigious. It is even mandatory for politicians to speak the local dialect in Bavaria. In particular, the Bavarian dialect of Germany holds a significant and independent position within the conceptual framework of languages (Adler, 2019). A survey revealed that the Bavarian dialect is considered the second most appealing German dialect (29,6%), after the Northern German dialect (34,9%), while only about 7% found the Berlin dialect attractive (Gärtig et al., 2010; Adler and Plewnia, 2018). At the same time, a mere 5% of respondents found the Berlin dialect unappealing, whereas having no dialect at all was rated as unattractive by 32,6% of the participants (Gärtig et al., 2010). Thus, to obtain a more nuanced understanding, it would be beneficial to conduct a comparative study involving multiple dialects as well as add an assessment of subjective dialect connotations. Moreover, as dialects are a means of positive identification within a group and signify a sense of attachment to a particular region (Wiese, 2012), varying levels of identification may exist among different dialects. This can affect the degree of perceived similarity and subsequently influence assessments of trustworthiness and competence.

Fourth, our study employed a video featuring NAO, a compact and intelligent-looking social robot. It remains uncertain if its appearance aligns with all the connotations linked to the Berlin dialect. Humans may link voices with robots, and a mismatch in this connection could result in diverse outcomes in their interaction (McGinn and Torre, 2019).

Finally, we consider the limitations of our methodology for data collection and data analysis. With regard to data collection, it will be important to provide converging evidence for this internet-based study by conducting both laboratory-based and real-life research in future projects. With regard to data analysis, more advanced modeling techniques, like linear mixed modeling, can offer greater flexibility compared to stepwise regression and can usefully be employed to uncover additional effects in our data, including further variability driven by participant characteristics.

Also, the topic of communication can influence the assessment of a robot that speaks a particular dialect. Using standard German would likely be more suitable for discussing a painting, while a dialect such as the Berlin dialect could be more appropriate for conversations about everyday events or work-related topics (*topic-based shifting*) (Walker, 2019).

An overall point for future investigations is that certain scholars view trust as a construct that has multiple dimensions. For example, Law and Scheutz (2021) differentiate between performance-based trust and relation-based trust. Future research on trust should take into account these different aspects and explore their implications in various contexts. Finally, objective measures of trust, for example, following a robot’s advice or task delegation should be used to better operationalize the outcome (Law and Scheutz, 2021).

Overall, our study provides valuable insights into how language proficiency and other demographic factors influence human-robot interaction and robot perception. Our results can inform the development of more effective robots that are tailored to meet the needs and expectations of diverse user groups. Further research is needed to explore the role of gender, age, and dialect in human-robot interaction and perception, and to identify additional factors that may influence trust and competence evaluation.

## Data Availability

The datasets presented in this study can be found in online repositories. The names of the repository/repositories and accession number(s) can be found below: https://osf.io/pfqg6/.

## Footnotes

1 The video material used was made and provided by Tristan Kornher, student at the University of Potsdam.

2 Multicollinearity was tested and rejected using VIF values ranging from 1.023 to 3.461 (substantially below the 10 threshold). Autocorrelation was absent, shown by Durbin-Watson statistics between 1.780 and 2.400 (within the acceptable range of 1.5–2.5). Normality of residuals was checked via P-P plots of standardized residuals.
