# Supplementary material for: “Ick bin een Berlina”: dialect proficiency impacts a robot’s trustworthiness and competence evaluation
Source: Front Robot AI. 2024 Jan 29;10:1241519. doi: 10.3389/frobt.2023.1241519 (PMC10859411; doi:10.3389/frobt.2023.1241519)
Supplement: Supplementary file 1 [file Table1.pdf]

Supplementary Table S1

Items for measuring trust towards the robot with translation.

Original items	Translation (Including the wording change)
The system is deceptive. (R)	Der Roboter ist trügerisch. (R)
The system behaves in an underhanded manner. (R)	Der Roboter verhält sich hinterhältig. (R)
The system's actions will have a harmful or injurious outcome. (R)	Die Handlungen des Roboters haben ein schädliches oder verletzendes Ergebnis. (R)
I am suspicious of the systems intent, action or outputs. (R)	Ich bin misstrauisch gegenüber der Absicht, der Handlung oder den Handlungsergebnissen des Roboters. (R)
I am wary of the system. (R)	Ich bin vorsichtig gegenüber dem Roboter. (R)
I can trust the system.	Ich kann dem Roboter vertrauen.
I am confident in the system.	Ich bin von dem Roboter überzeugt.
The system is reliable.	Der Roboter ist zuverlässig.
The system has integrity.	Der Roboter hat Integrität.
I am familiar with the system.	Ich bin mit dem Roboter vertraut.
The system is dependable.	Der Roboter ist verlässlich.
The system provides security.	Der Roboter bietet Sicherheit.

Note. (R) stands for an inverted item.
