# Supplementary material for: “Ick bin een Berlina”: dialect proficiency impacts a robot’s trustworthiness and competence evaluation
Source: Front Robot AI. 2024 Jan 29;10:1241519. doi: 10.3389/frobt.2023.1241519 (PMC10859411; doi:10.3389/frobt.2023.1241519)
Supplement: Supplementary file 2 [file Table2.pdf]

**Supplementary Table S2**

*Items for measuring the competence of the robot with translation.*

| Original items | Translation (Including the wording change) |
|---|---|
| Knowledgeable | Sachkundig |
| Interactive | Interaktiv |
| Responsive | Ansprechbar/empfänglich |
| Capable | Leistungsfähig |
| Competent | Kompetent |
| Reliable | Zuverlässig |
